# (Substituted-quinoline-1-yl) Methylbenzylammonium Chloride: Quaternization Reaction Process, Corrosion Inhibition Behavior, and Calculation Process

**DOI:** 10.3390/molecules30244782

**Published:** 2025-12-15

**Authors:** Jianing Tian, Roman Zinatullin, Jianhua Qian, Yanping Li, Xueming Kang, Junhua Li, Yanan Wang, He Huang, Jinjuan Xing

**Affiliations:** 1 School of Petrochemical Engineering, Liaoning Petrochemical University, Fushun 113001, China; t15841312343@163.com (J.T.); qianjh@lnpu.edu.cn (J.Q.); m18745874818@163.com (Y.L.); lijunhua0521@163.com (J.L.); wynants@163.com (Y.W.); huanghe@lnpu.edu.cn (H.H.); 2 Department of General, Analytical and Applied Chemistry, Ufa State Petroleum Technological University, 450064 Ufa, Russia; rim0812@mail.ru

**Keywords:** quinoline, quaternary ammonium salt, corrosion inhibitor, electro-chemical behavior, simulation calculation

## Abstract

In this paper, three quinoline-based quaternary ammonium salts were successfully synthesized, with quinoline, 8-hydroxyquinoline, and 8-methoxyquinoline used as raw materials, and benzyl chloride as a quaternization reagent. The as-synthesized quaternary ammonium salts showed excellent corrosion inhibition performance in the acidic environment based on the derivative’s cationic activity, heteroatom electron effect, and conjugated aromatic system. Specifically, the methoxy-containing product exhibited the highest corrosion inhibition efficiency of 96.92%. The quantitative calculation analysis demonstrated that the addition of methoxy and hydroxyl groups facilitated the quaternization reaction and enhanced the adsorption of the quaternary ammonium salts on the metal surface. The molecular dynamics study indicated that the three corrosion inhibitors adsorbed on the metal surface in a parallel orientation. This study has a certain reference significance for the research and development of quaternary ammonium salt as corrosion inhibitors in an acidic environment.

## 1. Introduction

Due to the mechanical processing properties, excellent electrical and thermal conductivities, and good corrosion resistance, copper (Cu) and Cu-based alloys are widely used in the chemical processing industry, shipbuilding engineering, electronics, and the energy industry. However, the presence of oxygen and some corrosive anions (such as chloride ions and carbonate ions) can lead to pitting corrosion on the copper surface in a highly acidic environment, which may have a negative impact on the performance of the copper-constructed system [1]. Therefore, preventing and controlling the Cu corrosion in acidizing solutions represents a critical and urgent challenge. Among various anti-corrosion strategies, the addition of corrosion inhibitors stands out as one of the most effective approaches for copper protection [2]. The common corrosion inhibitors are mostly organic compounds, which usually contain heteroatoms (N, S, O, P) and/or conjugated π bonds [3], such as imidazolines, quinolines, quaternary ammonium salts [4], and Mannich bases [5], etc. Among them, quinoline corrosion inhibitors exhibit excellent performance in metal corrosion protection due to their simple preparation process and low toxicity [6].

Ser’s group [7] established linear and nonlinear quantitative structure-property relationships (QSPR) based on mathematical models and verified the important role of quinoline as a corrosion inhibitor. Verma et al. [8] explored the corrosion inhibition performance of quinoline derivatives with different polar substituents at the 8-position on metals, such as 8-hydroxyquinoline (8-HQ), 8-methoxyquinoline (8-MQ), 8-aminoquinoline (8-AQ), and 8-nitroquinoline (8-NQ). The results indicated that the abovementioned derivatives exhibited good corrosion inhibition performance at a low concentration. Zhou Kun et al. [9] studied the anti-corrosion behavior of quinoline and 8-hydroxy-5-benzoquinoline derivatives. Punita Mourya et al. [10] discussed the corrosion inhibition performance of the hydroxyl group in marigold extracts on metals, pointing out that the extracts bound to metals with vacant d-orbitals through the conjugated π electrons in their aliphatic chains. The results demonstrated that the anti-corrosion performance of quinoline derivatives was excellent. Moreover, the introduction of substituents, such as hydroxyl, methoxy, and methyl groups, further improved their effectiveness as corrosion inhibitors for metals.

In order to enhance the surface activity and improve corrosion inhibition performance of quinoline derivatives in the characteristic system. Different from introducing quinoline substituents, Zong [11] used quinoline and pyridine as the main raw materials to react with benzyl chloride, epichlorohydrin, allyl chloride, dibromoethane, and *p*-chloromethylstyrene, respectively. Eight quinoline quaternary ammonium salt derivatives were synthesized. Their corrosion inhibition performances on carbon steel in an acidic system were evaluated. The results revealed that the above quinoline quaternary ammonium salts exhibited excellent corrosion inhibition performance. The author proposed that benzyl quinoline chloride could dissociate quaternary nitrogen cations with a larger molecular weight in an acid solution. The hydrophobic groups of quaternary nitrogen cations and the hydrophilic groups centered on nitrogen atoms gave the ions strong surface activity and allowed them to easily adsorb on the metal surface. In this study, quaternization reagents were systematically studied, indicating that quaternization was beneficial for improving the corrosion inhibition performance of quinoline compounds on metals.

Herein, this study discusses the quaternization process of different substituents and successfully synthesizes three quaternary ammonium salt derivatives with different substituents. The corrosion inhibition performance of the products in 1 M HCl solution was measured by potentiodynamic polarization experiments and electrochemical impedance tests; all three quaternary ammonium salts were mixed-type corrosion inhibitors, mainly for inhibiting cathodic corrosion reaction, and the (8-methoxyquinolin-1-yl) methylbenzylammonium chloride (OCH_3_-S) exhibited the best corrosion inhibition performance. The contact angle test indicated that the OCH_3_-S on the metal surface had the largest contact angle. The results of quantum chemical calculations and molecular dynamics simulations illustrated that the OCH_3_-S had the highest adsorption energy on the copper surface, which further demonstrated the strong adsorption effect of the corrosion inhibitor on the metal surface. It is believed that the results in this paper may provide more guidance for designing more high-performance inhibitors for preventing metal corrosion.

## 2. Results and Discussion

**Figure 1:**
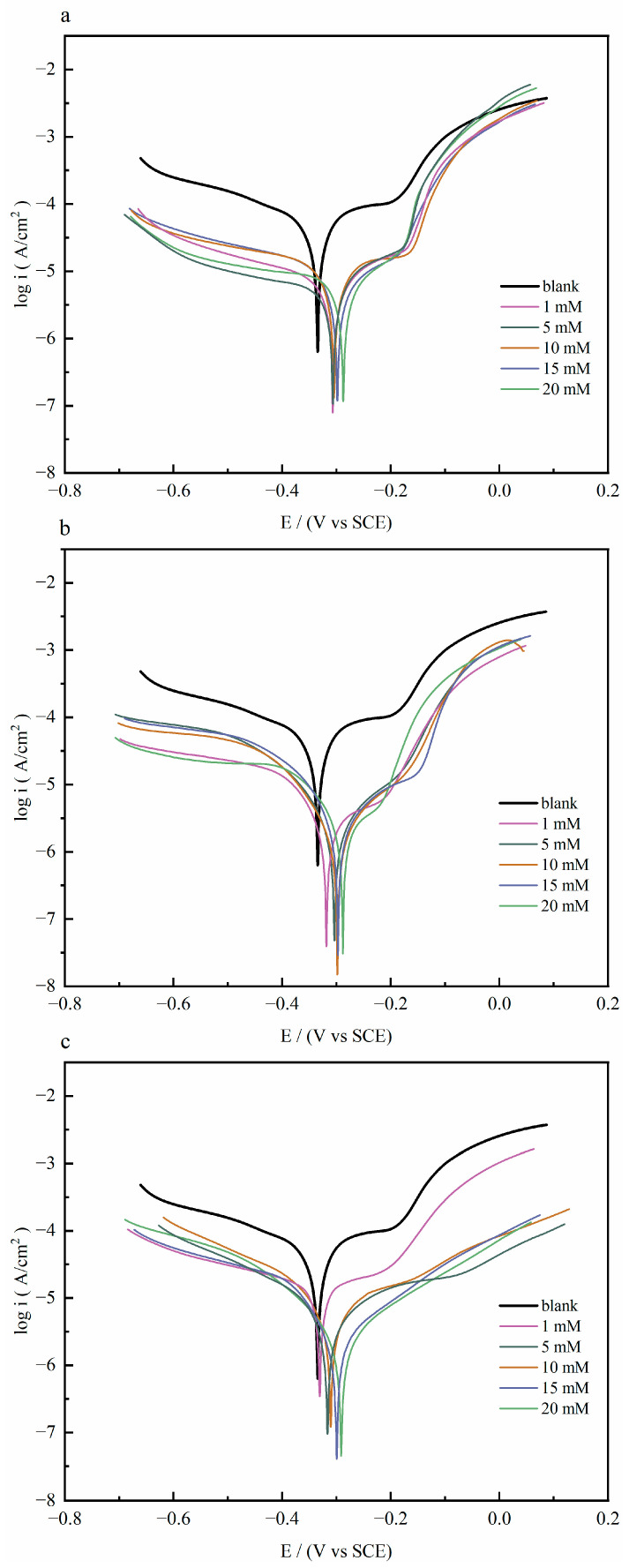
The polarization curves of copper with and without different concentrations of (**a**) Q-S, (**b**) HQ-S, and (**c**) OCH_3_-S in 1 M HCl solution at 298 K.

Figure 1 shows the polarization curves of copper sheets with and without different concentrations of quaternary ammonium salts in 1 M HCl solution at 298 K. The influence of the quaternary ammonium salt inhibitors on the dissolution process of the anode and the hydrogen ion reduction reaction at the cathode was investigated, respectively.

The electrochemical parameters, including corrosion current density (*I*_corr_), corrosion potential (*E*_corr_), and Tafel slope in cathode and anode (*β*c and *β*a), were obtained by extrapolating the linear Tafel section of the cathode curve and calculating the anode Tafel plot. The relevant electrochemical information and the calculated inhibition rates are shown in Table 1. The inhibition efficiency ($\eta_{p}$ %) was obtained by Equation (1) [12]; $I_{\mathrm{corr}}^{0}$ and $I_{\mathrm{corr}}$ (A·cm^−2^) represent the corrosion current densities without and with the addition of inhibitor molecules, respectively.

(1) $$\eta_{p}\%=\frac{I_{corr}^{0}-I_{corr}}{I_{corr}^{0}}\times 100$$

As shown in Table 1, the blank group that was without corrosion inhibitors exhibited the highest corrosion current density (2.1526 × 10^−5^ A·cm^−2^). When a quaternary ammonium salt was added, the corrosion current density reduced, resulting in a significant improvement in their corrosion inhibition performance. The polarization curves showed that the corrosion rate slowed on the anodic branch, and the current stabilized at a consistently low value. As the concentration of quaternary ammonium salt was 20 mM, the corrosion inhibition efficiency was the highest. The corrosion current densities of Q-S, HQ-S, and OCH_3_-S reduced to 1.6251 × 10^−6^ A·cm^−2^, 1.3406 × 10^−6^ A·cm^−2^, and 8.1103 ×10^−7^ A·cm^−2^. The inhibition efficiency ($\eta_{p}$) was in the order of OCH_3_-S (96.23%) < HQ-S (93.77%) < Q-S (92.45%).

Compared with the corrosion potential of the blank solution, the potential differences after adding inhibitors were all lower than ±85 mV, indicating that the three quaternary ammonium salts belonged to the mixed-type corrosion inhibitors, mainly inhibiting the cathodic and anodic reactions by adsorbing on the copper surface. Specifically, all of the cathodic branches of the Tafel curves were in parallel with each other, indicating that the mechanism of the cathodic reactions was not changed by the addition of these quinoline compounds. In the case of Q-S, its anodic branch was steep and overlapped with the curve of the blank solution until they intersected at approximately 0.11 V (vs. SCE), which indicated that the Q-S molecules gradually desorbed from the copper surface with increasing potential and were completely detached at around 0.11 V (vs. SCE). In contrast, the anodic curves of HQ-S and OCH_3_-S were observed to run parallel to that of the blank, indicating the formation of a more stable adsorption film on copper compared to Q-S [13].

**Figure 2:**
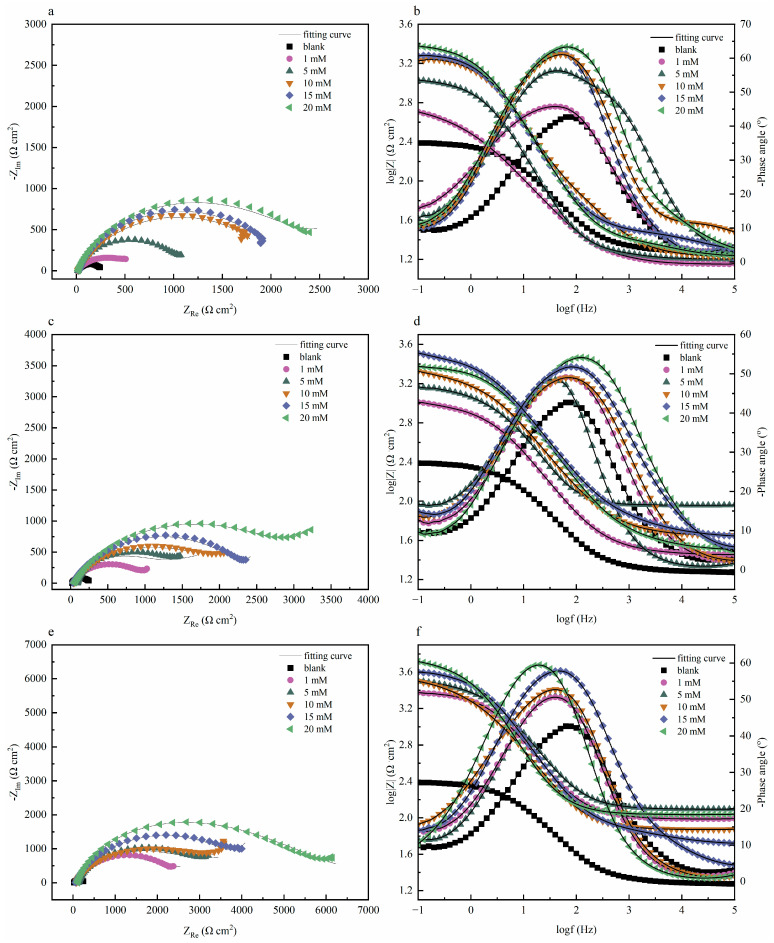
The Nyquist and Bode plots of copper with and without different concentrations of the investigated inhibitors in 1 M HCl solution at 298 K, (**a**,**b**) Q-S, (**c**,**d**) HQ-S, and (**e**,**f**) OCH_3_-S.

Electrochemical impedance tests were performed on systems with and without quaternary ammonium salts at 298 K, and the results are exhibited in Figure 2a,c. The figures indicate that quinoline quaternary ammonium salts greatly influenced the corrosion inhibition performance of the prepared Cu sheet. The diameter of the capacitive reactance in the presence of the corrosion inhibitor was significantly greater than that of the blank group, and the electrochemical impedance improved significantly with the increase in the corrosion inhibitor concentration [14]. The impedance diameter of three quaternary ammonium salts followed the sequence: OCH_3_-S > HQ-S > Q-S. The phase-angle Bode plots of three corrosion inhibitors are exhibited in Figure 2d,f. Only one time constant was presented in the Bode plot, thus further confirming that the corrosion behavior occurred due to the charge transfer process [15]. The phase angle shifted towards the high-frequency region due to the increase in corrosion inhibitor concentration, which was triggered by the microstructural variations in the metal-solution interface. The number of corrosion-inhibitor molecules adsorbed on the surface of the metal sheet increased, further enhancing the surface coverage and thus achieving a better corrosion inhibition [16]. The electrochemical impedance parameters of the three corrosion inhibitors (Q-S, HQ-S, and OCH_3_-S) are shown in Table 2.

**Figure 3:**
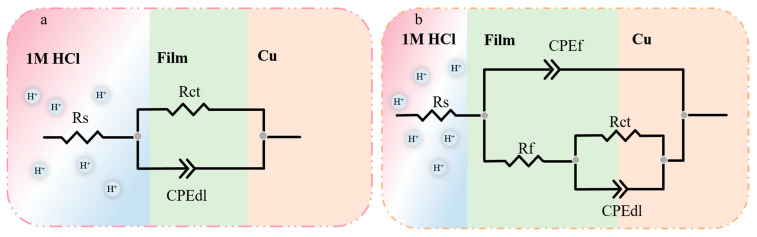
Electrical equivalent circuit used to model the interface copper in 1 M HCl solution (**a**) without and (**b**) with the corrosion inhibitor.

An equivalent circuit model was proposed by simulating the electrochemical impedance data (Figure 3). Here, *R_s_* stands for the solution resistance, *R_f_* represents the resistance of the protective inhibitor film formed on the copper surface, *R_ct_* is the charge transfer resistance, CPE_f_ and CPE_dl_ are the constant phase angle elements reflecting double-layer capacitance (*C_dl_*) and film capacitance (*C_f_*), respectively. The CPE was mathematically expressed as follows:

(2) $$E_{CPE}=\frac{1}{Y_{0}{\left(jw\right)}^{n}}$$

where $Y_{0}$ was the magnitude of the *CPE*, *j* was the imaginary root, *ω* was the angular frequency, and *n* represented the deviation from the ideal behavior, which lay between 0 and 1. The CPE could display an inductor (n = −1), resistor (n = 0), capacitor (n = 1), or Warburg impedance (n = 0.5). The impendence parameters gained from those equivalent circuits are listed in Table 2.

The values of *C_f_* and *C_dl_* were calculated from *Y*_0_ and n as follows:

(3) $$C=Y_{0\;}{\left(\omega_{max}\right)}^{n-1}$$

where *ω*_max_ = 2π*f*_max_ and *f*_max_ was the frequency at the maximum value of the imaginary component of the impedance plot [17].

Apparently, the values of *C_f_* and *C_dl_*, which could be expressed according to the following formulas, also exhibited a tendency to decrease with incremental concentrations of the compounds studied.

(4) $$C_{f\;}=\frac{F^{2}S}{4RT}$$

(5) $$C_{dl}=\frac{\varepsilon^{0}\varepsilon }{d}\;S$$

Accordingly, the decrease in *C_f_* could be ascribed to the increase in the coverage area of the protective film at higher inhibitor concentrations, which resulted in a lower exposed surface area of the copper electrode. Furthermore, as the concentration increases, more inhibitor molecules are adsorbed onto the copper surface. This process not only further diminished the exposed area but also lowered the local dielectric constant and increased the thickness of the electric double layer. These factors contributed to the reduction in the *C_dl_* values [18].

The *η* values of these quaternary ammonium salts for copper in 1 M HCl solution were calculated from the polarization resistance *R_p_* (*R_p_ = R_f_ + R_ct_*), according to the following Equation (6) [3]:

(6) $$\eta \%=\frac{R_{P}-R_{P,0}}{R_{P}}\times 100$$

where *R_P_* and *R_P,_*_0_ were the sum of film resistance and charge transfer resistance of the copper electrode with and without inhibitors, respectively.

Table 2 demonstrates that the addition of the three inhibitors improved *R_ct_* and *R_f_* values, which suggested the formation of the adsorption film on the copper surface that inhibited the charge transfer process [19]. As a result, the inhibition efficiency (*η*) increased with increasing inhibitor concentration. At the concentration of 20 mM, the *η* values for Q-S, HQ-S, and OCH_3_-S reached 91.85%, 94.74%, and 96.92%, respectively. The order of inhibition performance aligned with the results obtained from polarization measurements, confirming the effective protective role of these inhibitors against copper corrosion.

### 2.1. Surface Morphology Analysis

**Figure 4:**
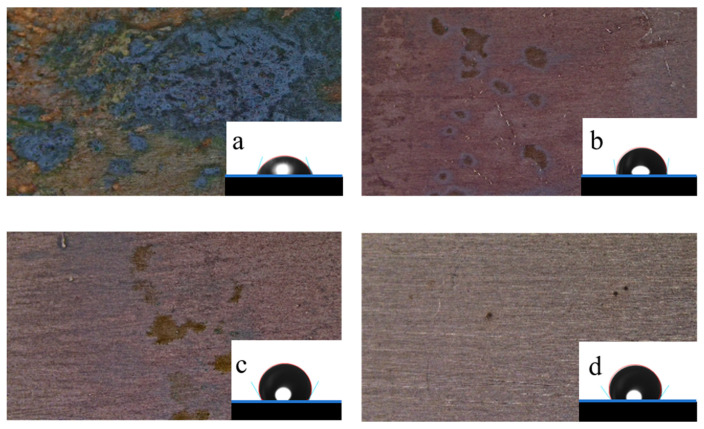
The contact angle images and corrosion morphologies of the metal surface without and with inhibitors in 1 M HCl solution at 298 K: (**a**) blank, (**b**) Q-S, (**c**) HQ-S, and (**d**) OCH_3_-S.

The hydrophilic–hydrophobic properties of the metal surface were analyzed with a contact angle measuring instrument at 298 K, and every sheet was tested five times at different test positions; the results are shown in Figure 4. The values of the test sheets were 72.99° (blank), 99.23° (Q-S), 119.89° (HQ-S), and 125.94° (OCH_3_-S), respectively, which illustrates that a hydrophobic membrane formed after adding different quaternary ammonium salts, thus inhibiting the metal sheet from corrosion [20]. Among the three corrosion inhibitors, the hydrophobic protective film formed by OCH_3_-S on the metal surface displayed the largest contact angle, indicating that the metal surface coated by OCH_3_-S was poorly wetted by the corrosive media [21]. The surface morphologies (Figure 4) also verified the existence of the protective film.

### 2.2. Quantum Chemical Calculation

**Figure 5:**
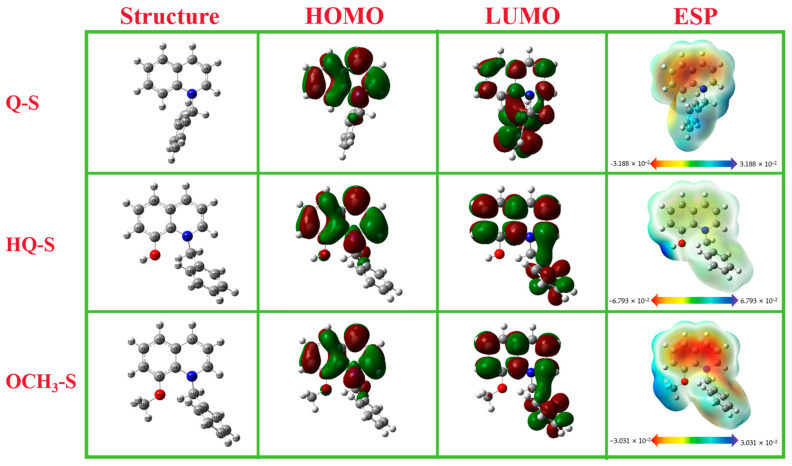
The optimized molecule structures, HOMO orbital distributions, LUMO orbital distributions, and electrostatic potential (ESP) maps of Q-S, HQ-S, and OCH_3_-S.

The relevant quantum chemical parameters of Q-S, HQ-S, and OCH_3_-S molecules are shown in Figure 5. The optimized molecular structures, HOMO orbital distributions, LUMO orbital distributions, and electrostatic potential (ESP) maps were listed, respectively. As illustrated in Figure 5, the orbital distributions of HOMO and LUMO were mainly concentrated near quinoline heterocycles. It is generally believed that the greatest electron density site of HOMO represents the electron-donating ability of the molecule, and the energy of LUMO indicates the greatest electron-drawing capability [22]. In the frontier orbital electron density distributions of the three molecules, the quinoline ring becomes the active site, showing excellent electron-donating ability, while the whole quaternary ammonium salt molecule could be employed as a good electron acceptor.

The local reaction active sites of corrosion inhibitors were studied by ESP distribution. The ESP distribution map consists of red and blue regions, where the red region represents a nucleophilic reaction site, and the blue region represents an electrophilic reaction site. The red region (positive electrostatic potential, electron-rich region) related to the nucleophilic reaction was mainly distributed around the quinoline ring, and the N and O atoms were the active centers with the strongest binding ability to the copper surface, which were more likely to form a coordination bond with the metal. The blue region (negative electrostatic potential, electron-deficient region) associated with the electrophilic reaction was obviously centered on -OH, -OCH_3_, and benzylic sites [23].

The quantum chemical parameters of Q-S, HQ-S, and O CH_3_-S are shown in Table 3. Here, *E_HOMO_*, *E*_LUMO_, Δ*E*, *χ*, *μ*, *γ*, and Δ*N* were calculated by the following Equations (7)–(12) [24]:

(7) $$\Delta E=E_{LUMO}-E_{HOMO}$$

(8) $$\chi =\frac{I+A}{2}$$

(9) $$\gamma =\frac{I-A}{2}$$

(10) $$\Delta N=\frac{\chi_{\mathrm{Cu}}-\chi_{inhibitor}}{2\left(\eta_{\mathrm{Cu}}-\eta_{inhibitor}\right)}$$

(11) $$I=-E_{HOMO}$$

(12) $$A=-E_{LUMO}$$

As shown in Table 3, the *E*_HOMO_ values of Q-S, HQ-S, and OCH_3_-S were −6.053 eV, −5.718 eV, and −5.633 eV, and the corresponding *E*_LUMO_ values were −1.464 eV (Q-S), −1.479 eV (HQ-S), and −1.416 eV (OCH_3_-S), respectively. The ΔE of the three corrosion inhibitors followed the sequence of OCH_3_-S < HQ-S < Q-S, which could be considered because OCH_3_-S was inclined to provide electrons to acceptors of low molecular orbital energy. In other words, 8-OCH_3_ showed the best corrosion inhibition performance.

The electron-withdrawing ability of a molecule or atom could be illustrated by the electronegativity (*χ*). As given in Table 3, the *χ* values of HQ-S (3.599 eV) and OCH_3_-S (3.525 eV) were all less than that of the copper (4.48 eV), which indicated that the electrons were absorbed on the surface of copper metal [25]. Compared with Q-S and OCH_3_-S, the value of OCH_3_-S exhibited the least *γ* value (2.109 eV), indicating that OCH_3_-S had the best electron-donating ability and the best corrosion inhibitor performance, which was consistent with the experimental results. The dipole moments (*μ*) contributed to the irregular distribution of charge in atoms. The inhibitor molecules exhibited higher dipole moments than H_2_O molecules, which could promote stronger dipole interactions with the metal surface, thus showing better corrosion inhibition performance. The dipole moments of Q-S, HQ-S, OCH_3_-S, and H_2_O were 2.012 Debye, 1.550 Debye, 2.109 Debye, and 1.88 Debye, which indicated that the three inhibitors could easily replace H_2_O molecules to adsorb on the copper surface, and OCH_3_-S was the most effective. Δ*N* represented the flow direction between two different electronegativity systems. In general, the value of Δ*N* more than zero signified that electrons were transferred from the corrosion inhibitor molecule to the metal surface and vice versa. The value of Q-S was 0.183, which demonstrated the electron transfer orientation. The transfer direction of the other two quaternary ammonium salts was also the same, that is, from the corrosion inhibitors to the metal [26].

### 2.3. Molecular Dynamics Simulation Process

**Figure 6:**
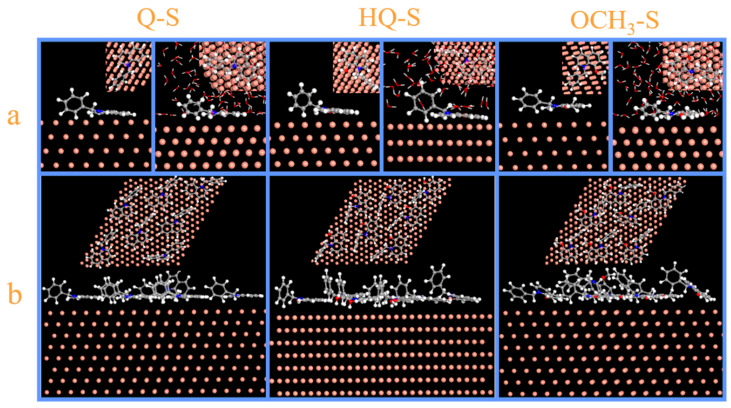
The dynamic simulation images of Q-S, HQ-S, and OCH_3_-S on Cu (111) plane: (**a**) the side and top view of a single-molecule adsorption configuration in the vacuum and aqueous system, and (**b**) the side and top view of a multi-molecule adsorption configuration in the vacuum system.

The vacuum and aqueous system models were established to analyze the adsorption processes of Q-S, HQ-S, and OCH_3_-S. Figure 6 exhibited the side- and top-view diagrams of three corrosion inhibitor molecules optimized on the Cu (111) surface under vacuum and H_2_O molecule systems. According to these images, the single molecule tended to achieve parallel adsorption on the metal surface.

The multi-molecule adsorption configuration in the vacuum system is displayed in Figure 6b, in which the quinoline rings were oriented to maximize contact with the metal surface to form a dense protective film, effectively blocking the destruction of the copper sheet by corrosive media, thereby protecting the metal.

The adsorption dynamics of the inhibitors were conducted on the Cu (111) plane in a system filled with H_2_O molecules, and the results were displayed in Figure 6a. All three inhibitor molecules exhibited competitive adsorption with H_2_O molecules and achieved parallel adsorption on the metal surface. The adsorption energy calculation of the metal-inhibitor interface was displayed in the following Equation (13):

(13) $$E_{\mathrm{adsorption}}=E_{\mathrm{total}}-(E_{\mathrm{surface}+\mathrm{solution}}+E_{\mathrm{inhibitor}})$$

where *E*_total_ (kJ·mol^−1^) was the energy of the system, *E*_surface+solution_ (kJ·mol^−1^) was the total energy of Cu (111) and water molecules, and *E*_inhibitor_ (kJ·mol^−1^) was the energy of corrosion inhibitors.

In a system with water molecules filling the vacuum space, the calculated adsorption energies of Q-S, HQ-S, and OCH_3_-S on copper were −471.24, −538.77, and −588.94 kJ/mol, respectively. Among them, OCH_3_-S exhibited the highest adsorption energy, indirectly indicating its strongest adsorption capability on the metal substrate [27].

The local molecule reactivity could be characterized by the Fukui index. In general, the ideal electron-withdrawing site displays as the active site ($f_{k}^{+}$) with the larger nucleophilicity attack index, while the ideal electron-donating site serves as the active site ($f_{k}^{-}$) with a larger electrophilic attack index ($f_{k}^{-}$). The specific atom in the quinoline heterocyclic molecule, combining with the copper atom, was figured, in which the definition of the Fukui index was illustrated in the following Equations (14) and (15) [28]:

(14) $$f_{k}^{+}=q_{k}\left(N+1\right)-q_{k}\left(N\right)$$

(15) $$f_{k}^{-}=q_{k}\left(N\right)-q_{k}\left(N-1\right)$$

**Figure 7:**
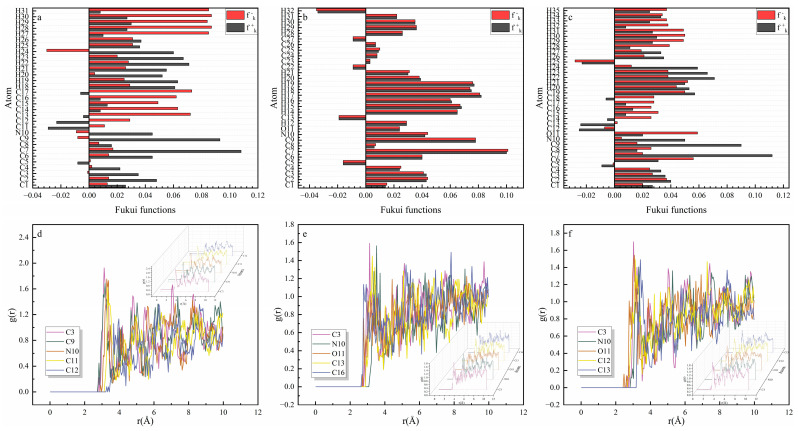
The Fukui index of (**a**) Q-S, (**b**) HQ-S, and (**c**) OCH_3_-S and the radial distribution functions of (**d**) Q-S, (**e**) HQ-S, and (**f**) OCH_3_-S.

In Figure 7a–c, the nitrogen, oxygen, and carbon atoms in the benzene ring of quinoline and its derivatives displayed larger $f_{k}^{+}$ and $f_{k}^{-}$ values, which were more likely to form a coordination bond with the copper and were involved in the chemical adsorption process.

The radial distribution function (RDF) was used to investigate the interaction between the metal and the corrosion inhibitors. As presented in Figure 7d–f, the adsorption behavior of the corrosion inhibitor molecules was determined by the first peak. The calculation results indicated that the distance between the oxygen atoms of the inhibitors and copper was less than 3.5 Å, confirming the chemical adsorption of these inhibitors into the copper surface.

### 2.4. Adsorption Isotherm Models

**Figure 8:**
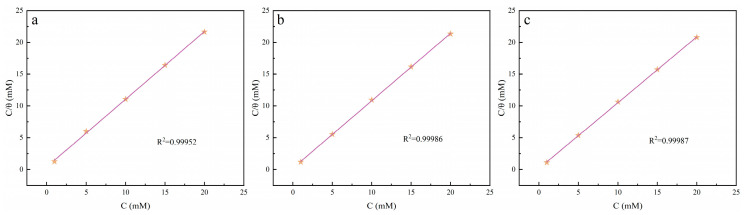
The adsorption isotherm model fitted with polarization data for (**a**) Q-S, (**b**) HQ-S, and (**c**) OCH_3_-S on the surface of copper at 298 K.

It is well recognized that the adsorption of inhibitor molecules at the metal/solution interface depends on the inhibitor’s chemical composition, the temperature, and the electrochemical potential at the metal/solution interface. In fact, the solvent H_2_O molecules can also adsorb at the metal/solution interface. Therefore, the adsorption of inhibitor molecules from the aqueous solution could be regarded as a quasi-substitution process between the inhibitors and water molecules at the interface:

Inh._(sol)_ + xH_2_O_(ads)_ ↔ Inh._(ads)_ + xH_2_O_(sol)_ (16)

where x was the number of water molecules replaced by one inhibitor. Basic information on the interaction between the inhibitor and the copper surface could be provided by the adsorption isotherm. The best fit was obtained with the Langmuir isotherm, which could be expressed by the following Equation (17):

(17) $$\frac{C_{inh}}{\theta }=\frac{1}{K_{ads}}+C_{inh}$$

where θ (θ = *ηp*%/100) represented the degree of surface coverage measured at 298 K, *K_ads_* was the adsorption/desorption equilibrium constant, *C_inh_* was the inhibitor concentration, and *ηp* represented the inhibition efficiency obtained from polarization measurements. The results in Figure 8 displayed a significant linear relationship that existed between C/θ and C at 298 K.

The standard adsorption free energy $\Delta G_{\mathrm{ads}}^{0}$ was calculated according to the following Equation (18):

(18) $$\Delta G_{\mathrm{ads}}^{0}=-\mathrm{RTln}\left(55.5K_{ads}\right)$$

where R was the universal gas constant, T the thermodynamic temperature, and the value of 55.5 was the concentration of water in the solution.

When the $\Delta G_{\mathrm{ads}}^{0}$ value was greater than or equal to −20 kJ/mol, the adsorption mechanism was physical adsorption, whereas the value was less than or equal to −40 kJ/mol indicating chemical adsorption. In this study, the calculated values of $\Delta G_{\mathrm{ads}}^{0}$ for Q-S, HQ-S, and OCH_3_-S were −28.13, −28.73, and −28.98 kJ/mol, respectively, indicating that the tested inhibitor involved both physical and chemical adsorption. These negative values indicated the spontaneous process of inhibitors adsorbing onto the surface of copper metal, and the stability of the adsorption layer [29].

## 3. Experimental Methods

### 3.1. Materials

Quinolone (99.9%, AR), 8-hydroxyquinoline (99.9%, AR), 8-methoxyquinoline (99.9%, AR), and benzyl chloride (99.9%, AR) were purchased from Shanghai Macklin Biochemical Co., Ltd. (Shanghai, China). Acetonitrile (99.9%, AR), methanol (99.9%, AR), and methylene dichloride (99.9%, AR) were purchased from Liaoning Quanrui Reagent Co., Ltd. (Shenyang, China). Deionized (DI) water (18.2 MΩ cm^−1^) was used in this experiment. Hydrochloric acid (HCl) was purchased from Chengdu Kelong Chemical Co., Ltd. (Chengdu, China). The pure copper specimen (25 × 25 × 2 mm^3^, Cu > 99.9%) was purchased from Jiangsu Taizhou Jumi New Materials Co., Ltd. (Taizhou, China). The 800-, 1200-, and 2000-grit sandpapers were obtained from Zhejiang Yiwu Yufei Trading Co., Ltd. (Yiwu, China).

### 3.2. Physicochemical Characterization

Fourier Transform Infrared Spectroscopy (FT-IR) was performed using a Bruker INVENIO-S spectrometer (Bruker, Billerica, MA, USA) with the KBr pellet method. The spectra were acquired over a wavelength range of 400–4000 cm^−1^. Nuclear Magnetic Resonance Spectroscopy (NMR) spectra of quinoline quaternary ammonium salts in deuterated DMSO-*d*_6_ were recorded using TMS as an internal standard on an instrument (AVANCE III HD 400M, Bruker, USA), with δ values in ppm and *J* values in Hz. A Multi Autolab M204 electrochemical station (Metrohm, Herisau, Switzerland) was used for the experiment. A contact angle measuring instrument (SDC-100, Shengding Precision Instruments Co., Ltd., Dongguan, China) was used to evaluate the hydrophilic/hydrophobic properties of all the inhibitors on the metal surface. All the samples were observed using a stereomicroscope (SMZT45T, Sunjoy Instrument Co., Ltd., Beijing, China).

### 3.3. Electrochemical Measurements

The copper sheet was polished with 800-, 1200-, and 2000-grit sandpapers in turn, then cleaned with ethanol and deionized water, leaving a 1 × 1 cm^2^ area exposed to the aggressive solution for the electrochemical test. All electrochemical tests were performed using a conventional three-electrode system, with the experimental copper sheet as the working electrode, a saturated calomel electrode as the reference electrode, and a platinum sheet (1 × 1 cm^2^) as the counter electrode. The aggressive solution was prepared by using a 1 M HCl solution without and with various concentrations (1, 5, 10, 15, and 20 mM) of inhibitors. All the tests were carried out at 298 ± 0.5 K using a temperature-controlled water bath under unstirred conditions.

To ensure the steady state of the electrochemical measurement, an open-circuit potential (OCP) test for 2000 s was conducted before the experiment. Then, the Nyquist and Bode plots were acquired through the electrochemical impedance spectroscopy (EIS) measurements with a 20 mV peak-to-peak sine wave in the frequency range of 0.1 Hz to 100 kHz. Furthermore, the EIS test data were fitted and analyzed by Z-View 2.7 software. The polarization tests were carried out in the potential range of ±0.3 V vs. the E_OCP_ at a scan rate of 1 mV s^−1^ [30].

### 3.4. Calculation Details

The quantum chemical calculations of quinoline-based quaternary ammonium salt and other organic molecules in this work were performed using the Gaussian 06 software [31]. The geometric structure of molecules was optimized by employing density functional theory (DFT) in combination with the B3LYP/6-311G basis set (d). Then, some quantum chemical parameters of Q-S, HQ-S, and OCH_3_-S, including the energy of the highest occupied molecular orbital (*E*_HOMO_), energy of the lowest unoccupied molecular orbital (*E*_LUMO_), energy gap Δ*E* (Δ*E* = *E*_LUMO_ − *E*_HOMO_), electronegativity (χ), dipole moments (μ), absolute hardness (γ), and electron transfer parameters (ΔN), were calculated accurately [17].

In this work, molecular dynamics (MD) simulations were performed using the Materials Studio (MS, 2023) software from Accelrys Inc. (San Diego, CA, USA) [31]. Initially, the corrosion inhibitor molecules were optimized using the Dmol3 module. Then, a box (20 Å × 35 Å × 20 Å) was modeled to simulate the vacuum and aqueous environment in the Forcite and Amorphous Cell modules [32]. The binding energy between the corrosion inhibitor molecules and the Cu (111) surface was estimated through the MD process using the COMPASS force field. The simulations were performed in an NVT ensemble at 298 K, with a time step of 1 fs and a simulation time of 2000 ps [33].

### 3.5. Synthesis of Quinoline-Based Quaternary Ammonium Salts

The synthesis process of (8-hydroxyquinoline-1-yl) methylbenzyl ammonium chloride (HQ-S) was used to describe the typical preparation of quinoline-based quaternary ammonium salts. The molar ratio of 8-HQ to benzyl chloride was 1:1.2 throughout the experiment. First, 8-HQ was dissolved in an appropriate amount of acetonitrile. Subsequently, the solution was heated to 70 °C and maintained for 1 h under magnetic stirring. Then benzyl chloride was added dropwise to the preheated solution. After the addition was completed, the temperature was raised to 85 °C, and the reaction was allowed to proceed for 24 h. When the reaction ended, a small amount of solvent in the mixture was evaporated under low pressure, then the residual reactants were extracted with dichloromethane, and the mixed solution was filtered to obtain an insoluble precipitate. The precipitate was further purified by column chromatography (DCM:MeOH = 95:5) to obtain the product HQ-S. HQ-S (yellow solid): ^1^H NMR (400 MHz, DMSO): δ: 12.22 (1H, s, O11-H), 9.54 (1H, d, *J* = 5.9 Hz, C7-H), 9.25 (1H, d, *J* = 8.4 Hz, C9-H), 8.15 (1H, dd, *J* = 8.4, 5.7 Hz, C8-H), 7.89 (1H, dd, *J* = 8.1, 1.4 Hz, C1-H), 7.82 (1H, t, *J* = 7.9 Hz, C6-H), 7.68 (1H, dd, *J* = 7.8, 1.4 Hz, C2-H), 7.34 (3H, dd, *J* = 11.7, 7.2 Hz, C24-H, C25-H and C26-H), 7.18 (2H, d, *J* = 6.8 Hz, C23-H and C27-H), 6.62 (2H, s, C13-H). ^13^C NMR spectral data (100 MHz, DMSO) δ 152.2, 150.6, 150.0, 148.7, 137.0, 132.9, 132.8, 131.1, 129.3, 128.6, 126.9, 122.5, 121.5, 120.8, 65.87. FT-IR: ν(-OH), 3427 cm^−1^, ν(C-H) 3060 cm^−1^, ν(C=C) 1593–1544 cm^−1^, δ(C-N) 1360–1298 cm^−1^, ν(C-N) 1448 cm^−1^, δ(C-H) 845–739 cm^−1^.

**Figure 9:**
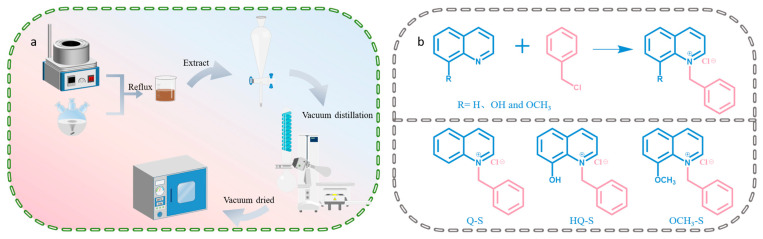
(**a**) The synthesis route of quinoline-based quaternary ammonium salts and (**b**) the chemical reaction formula.

The synthesis process of (quinolin-1-yl) methylbenzylammonium chloride (Q-S) and (8-methoxyquinolin-1-yl) methylbenzylammonium chloride (OCH_3_-S) was the same as above, using the corresponding substituted quinoline instead. The experimental schematic illustration is presented in Figure 9a,b.

Q-S (light brown solid): ^1^H NMR (400 MHz, DMSO) δ 9.87 (1H, d, *J* = 5.8 Hz, C7-H), 9.41 (1H, d, *J* = 8.3 Hz, C9-H), 8.57–8.50 (2H, m, C2-H, and C8-H), 8.31 (1H, dd, *J* = 8.4, 5.8 Hz, C3-H), 8.22 (1H, t, *J* = 8.0 Hz, C6-H), 8.03 (1H, t, *J* = 7.6 Hz, C1-H), 7.44–7.33 (5H, m, C12-H, C14-H, C15-H, C16-H, and C17-H), 6.43 (2H, s, C11-H). ^13^C NMR (100 MHz, DMSO): δ 151.0, 148.7, 138.0, 136.2, 134.4, 131.4, 130.5, 129.6, 129.25, 127.8, 123.0, 122.3, 119.8, 60.3. FT-IR: ν(-OH), 3448 cm^−1^, ν(C-H) 3060 cm^−1^, ν(C=C) 1593–1544 cm^−1^, δ(C-N) 1450 cm^−1^, ν(C-N) 1355–1300 cm^−1^, δ(C-H) 889–732 cm^−1^.

OCH_3_-S (yellow solid): ^1^H NMR (400 MHz, DMSO): δ 9.66 (1H, dd, *J* = 5.9, 1.5 Hz, C7-H), 9.35 (1H, dd, *J* = 8.4, 1.4 Hz, C1-H), 8.27 (1H, dd, *J* = 8.4, 5.8 Hz, C9-H), 8.05 (1H, d, *J* = 8.1 Hz, C6-H), 7.94 (1H, t, *J* = 8.0 Hz, C2-H), 7.72 (1H, d, *J* = 8.0 Hz), 7.40–7.28 (3H, m, C16-H, C17-H, and C18-H), 7.13 (2H, d, *J* = 6.7 Hz, C15-H, and 19-H), 6.53 (2H, s, C13-H), 3.89 (3H, s, C12-H). ^13^C NMR (100 MHz, DMSO) δ 153.0, 150.8, 148.9, 136.7, 132.7, 131.0, 130.1, 129.9, 129.8, 129.3, 128.5, 126.5, 124.2, 123.1, 117.2, 65.8, 57.5. FT-IR: ν(-OH), 3327 cm^−1^, ν(C=C) 1593–1536 cm^−1^, δ(C-N) 1375 cm^−1^, ν(C-N) 1177 cm^−1^, δ(C-H) 886–754 cm^−1^.

The related spectra of Q-S, HQ-S, and OCH_3_-S, including FT-IR, ^1^H NMR, and ^13^C NMR, were provided in Figures S1–S9.

## 4. Anti-Corrosion Mechanism

**Figure 10:**
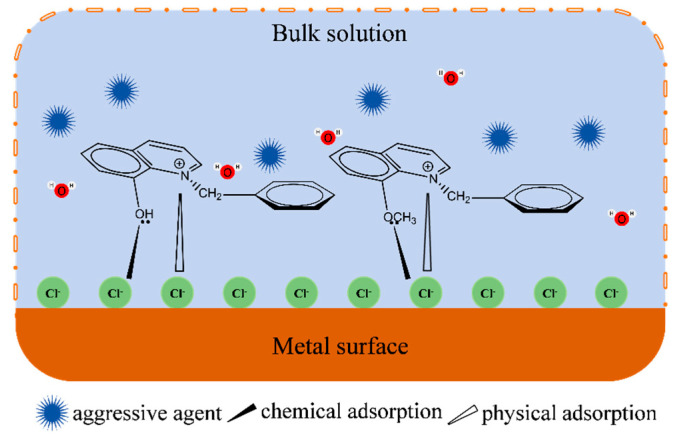
Adsorption mechanism of quaternary ammonium salt on metal surface.

Combined with the above experiment and calculation analyses, this paper provides the anti-corrosion mechanism of quaternary ammonium salt in an acid solution on the metal surface, and the mechanism is depicted in Figure 10. In an acidic medium, the metal surface was prone to dissolution, and the cation diffused into the medium. The metal surface enriched with electrons, or the adsorption of the chloride ion in the medium, could also make the metal surface present a negative state. The quaternary nitrogen cation quickly adsorbs on the metal surface through electrostatic interaction. This process could not only balance the charge of the metal surface and reduce the reactivity of the acid ions with the metal but also form a hydrophobic protective film that prevented the metal from erosion by the medium. Quinoline-based quaternary ammonium salts had strong surface activity, which enabled them to competitively adsorb on the metal surface. Moreover, the rich π electron cloud in the molecule could form coordination bonds with the metal, and the hydrophobic groups around the bonding atoms could stably adsorb on the metal surface. The adsorption film had a shielding effect, thus preventing the metal from corrosion [34].

## 5. Conclusions

In summary, the quaternization processes of quinoline derivatives were studied, and three quinoline-based quaternary ammonium salts were successfully prepared. The experiment indicated that the electron-donating substituents were favorable for the quaternization processes. The corrosion inhibition performance of the three quaternized products was studied, and the results indicated that the corrosion inhibition efficiency peaked at the concentration of 20 mM, and the corrosion inhibition performance of the three inhibitors followed the order: Q-S < HQ-S < OCH_3_-S. All three inhibitors functioned primarily as cathode-predominant, mixed-type inhibitors. The electron-donating effects of the methoxy and hydroxy groups enhanced the nucleophilicity of the nitrogen atom, which facilitated the SN_2_ reaction with benzyl chloride. By combining the methoxy and hydroxyl functionalities, the inhibitor molecule stably adsorbed on the metal surface. The quantum chemical calculations and molecular dynamics simulation further explained the excellent corrosion inhibition performance of methoxy and hydroxyl-substituted compounds on metals. Moreover, due to the electronic effect of the methoxy group, which is more conducive to adsorption on metal substrates, OCH_3_-S exhibited the best corrosion inhibition performance. This systematic investigation provides a deep understanding of how substituents’ electron-donating effects enhance copper corrosion inhibition performance and offers guidance for designing novel quaternary ammonium salt derivatives as efficient potential inhibitors.

## Figures and Tables

**Table 1:** Potentiodynamic polarization curves of Cu with and without inhibitors in 1 M HCl at 298 K.

Corrosion Inhibitor	*C* mM	*I*_corr_ A·cm^−2^	*E*_corr_ V	*β*a V·dec^−1^	−*βc* V·dec^−1^	*ηp* %
Blank	0	2.1526× 10^−5^	−0.34889	0.08388	0.06618	——
Q-S	1	4.1545 × 10^−6^	−0.30616	0.07125	0.06139	80.70
	5	3.5549 × 10^−6^	−0.30565	0.07150	0.06840	83.49
	10	2.0735 × 10^−6^	−0.30513	0.07504	0.07399	90.37
	15	1.8562 × 10^−6^	−0.29799	0.06011	0.06982	91.38
	20	1.6251 × 10^−6^	−0.28749	0.05258	0.07104	92.45
HQ-S	1	3.0785 × 10^−6^	−0.34039	0.09234	0.06853	85.70
	5	2.0665 × 10^−6^	−0.31612	0.08294	0.07146	90.40
	10	1.8541 × 10^−6^	−0.31092	0.11100	0.07277	91.39
	15	1.5353 × 10^−6^	−0.29954	0.09374	0.07732	92.87
	20	1.3406 × 10^−6^	−0.29171	0.08697	0.06592	93.77
OCH_3_-S	1	1.7866 × 10^−6^	−0.31597	0.11879	0.07518	91.70
	5	1.4376 × 10^−6^	−0.30185	0.10981	0.06692	93.32
	10	1.2667 × 10^−6^	−0.29784	0.10945	0.06442	94.12
	15	1.0071 × 10^−6^	−0.29585	0.09887	0.06726	95.32
	20	8.1103 × 10^−7^	−0.28963	0.08090	0.07251	96.23

**Table 2:** The fitting impedance data of Q-S, HQ-S, and OCH_3_-S.

Corrosion Inhibitor	*C* mM	*R_f_* Ω cm^2^	*R_ct_* Ω cm^2^	*R_p_* Ω cm^2^	*C_f_* μF cm^−2^	n_1_	*C_dl_* μF cm^−2^	n_2_	*η* %
blank	—	—	240	240	—	—	334.439	0.72	—
Q-S	1	19.0	1370	1389.0	121.670	0.85	118.439	0.70	82.48
	5	40.6	1484	1524.6	31.810	0.79	87.793	0.66	83.83
	10	209.0	2397	2606.0	28.127	0.77	23.236	0.60	89.99
	15	210.2	2432	2642.2	20.176	0.74	19.405	0.49	90.13
	20	220.8	2944	3164.8	14.799	0.71	14.385	0.44	91.85
HQ-S	1	72.2	1411	1483.2	43.412	0.86	188.526	0.49	83.99
	5	68.4	2447	2515.4	17.015	0.74	15.447	0.46	90.19
	10	236.6	2725	2961.6	12.505	0.73	11.258	0.46	91.19
	15	106.9	3076	3182.9	11.635	0.71	9.674	0.43	91.20
	20	309.7	4567	4876.7	10.632	0.69	8.222	0.63	94.74
OCH_3_-S	1	189.2	3163	3352.2	18.843	0.89	37.255	0.59	92.41
	5	67.0	4848	4915.0	11.801	0.87	8.873	0.41	95.05
	10	98.6	5655	5753.6	9.994	0.81	7.255	0.50	95.76
	15	29.0	6978	7007.0	8.981	0.80	6.886	0.59	96.56
	20	409.7	7787	8196.7	8.425	0.75	6.297	0.83	96.92

**Table 3:** The quantum chemical calculation results of the three corrosion inhibitors.

Corrosion Inhibitor	*E_LUMO_* (eV)	*E_HOMO_* (eV)	Δ*E* (eV)	*χ* (eV)	*γ* (eV)	Δ*N*	*μ* (Debye)
Q-S	−1.464	−6.053	4.589	5.321	2.295	0.183	2.012
HQ-S	−1.479	−5.718	4.239	3.599	2.120	−0.122	1.550
OCH_3_-S	−1.416	−5.633	4.217	3.525	2.109	−0.136	2.109

## Data Availability

The original contributions presented in this study are included in the article and Supplementary Materials. Further inquiries can be directed to the corresponding author.

## Supplementary Materials

The following supporting information can be downloaded at: https://www.mdpi.com/article/10.3390/molecules30244782/s1, Figure S1: ^1^H NMR spectra of Q-S; Figure S2: ^13^C NMR spectra of Q-S; Figure S3: FT-IR spectra of Q-S; Figure S4: ^1^H NMR spectra of HQ-S; Figure S5: ^13^C NMR spectra of HQ-S; Figure S6: FT-IR spectra of HQ-S; Figure S7: ^1^H NMR spectra of OCH_3_-S; Figure S8: ^13^C NMR spectra of OCH_3_-S; Figure S9: FT-IR spectra of OCH_3_-S.
