# Supplementary material for: (Substituted-quinoline-1-yl) Methylbenzylammonium Chloride: Quaternization Reaction Process, Corrosion Inhibition Behavior, and Calculation Process
Source: Molecules. 2025 Dec 15;30(24):4782. doi: 10.3390/molecules30244782 (PMC12735674; doi:10.3390/molecules30244782)
Supplement: Supplementary file 1 [file molecules-30-04782-s001.zip › molecules-4026935-supplementary.pdf]

Supplementary Material

(Substituted-quinoline-1-yl) methylbenzylammonium chloride: Quaternization reaction process, corrosion inhibition behavior and calculation process

Jianing Tian [1], Zinatullin Roman [2], Jianhua Qian [1],Yanping Li [1], Xueming Kang [1,*],

Junhua Li [1], Yanan Wang [1], He Huang [1], Jinjuan Xing [1,*]

（1. School of Petrochemical Engineering, Liaoning Petrochemical University,

Fushun, Liaoning 113001, China

2. Ufa State Petroleum Technological University, Ufa, 450064, Russian Federation）

Correspondence to: Xueming Kang (**kangxueming@lnpu.edu.cn**),Jinjuan Xing (**xingjinjuanln@163.com**)

Figure S1-S3 (Q-S)

Figure S1: ^{1}H NMR spectra of Q-S (400 MHz, DMSO-d_6)

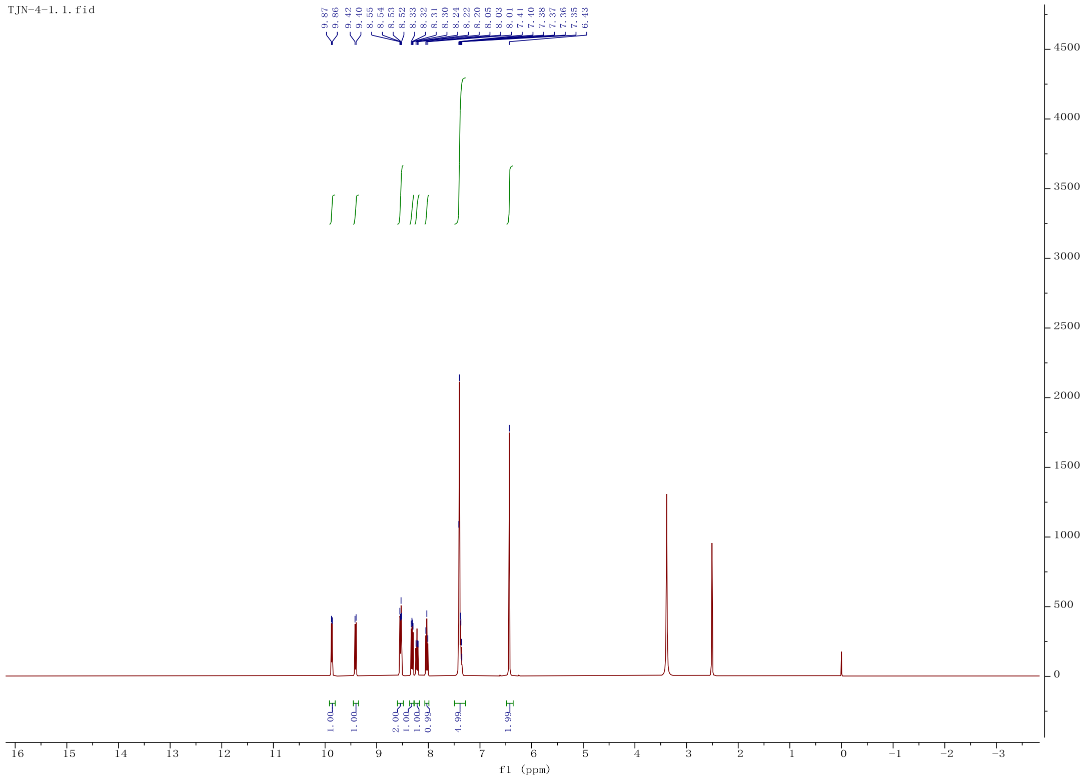

(Q-S) ^{1}H NMR (400 MHz, DMSO-d6, TMS) δ ppm： 9.87 (d , J = 5.8 Hz, 1H), 9.41 (d, J = 8.3 Hz, 1H), 8.57-8.50 (m, 2H), 8.31 (dd, J = 8.4, 5.8 Hz, 1H), 8.22 (t, J = 8.0 Hz, 1H), 8.03 (t, J = 7.6 Hz, 1H), 7.44-7.33 (m, 5H), 6.43 (s, 2H).

Figure S2: ^{13}C NMR spectra of Q-S (100 MHz, DMSO-d_6)

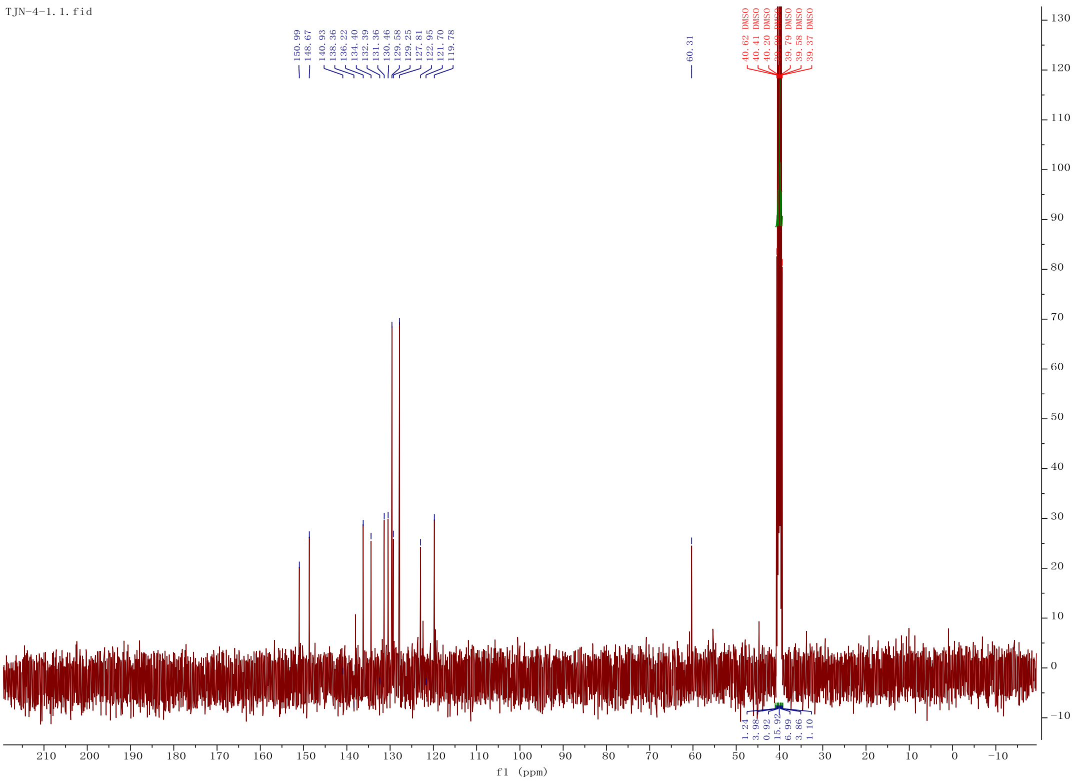

(Q-S) ^{13}C NMR (100 MHz, DMSO-d6) δ ppm. ^{13}C NMR (100 MHz, DMSO-d6) δ ppm 151.0, 148.7, 138.0, 136.2, 134.4, 131.4, 130.5, 129.6, 129.25, 127.8, 123.0, 122.3, 119.8, 60.3.

Figure S3: FT-IR spectra of Q-S

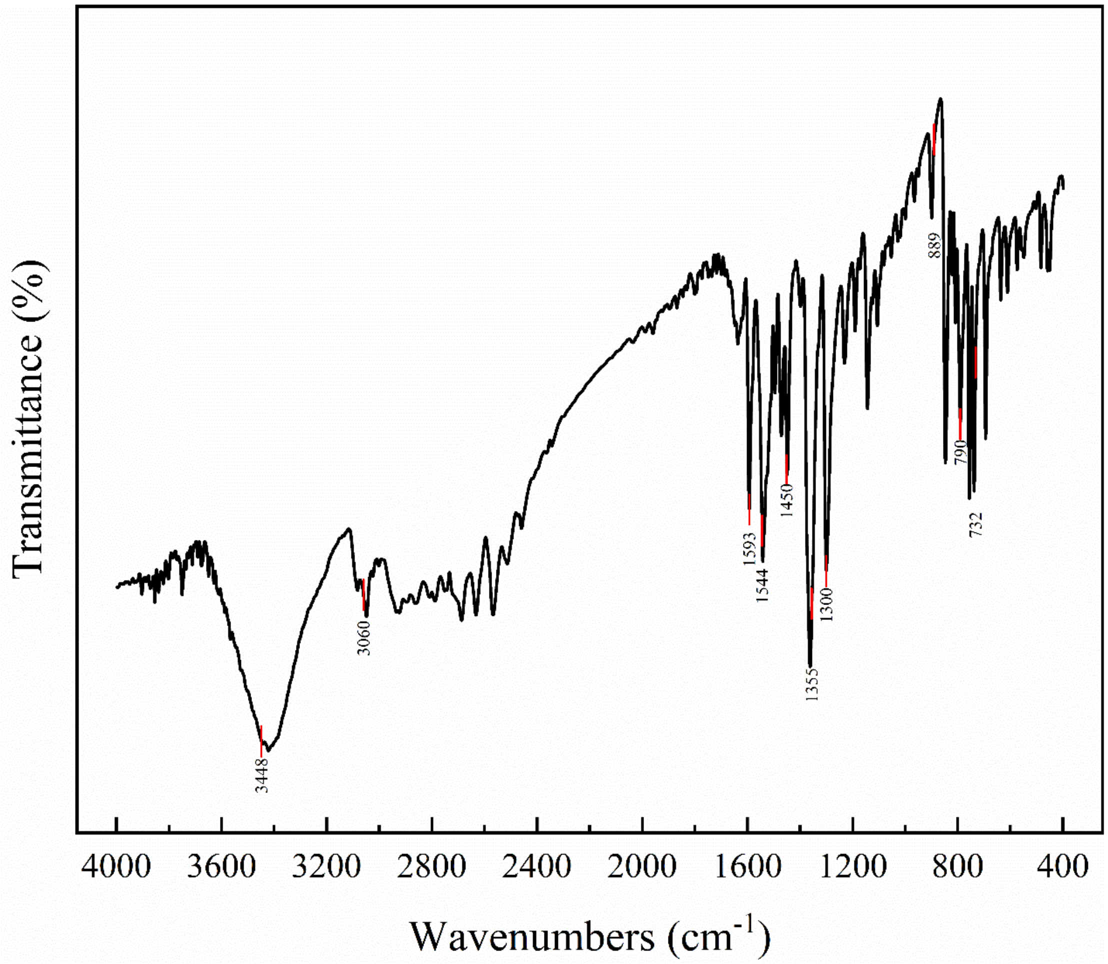

(Q-S) FTIR. ν(-OH), 3448 cm^{-1}, ν(C-H) 3060 cm^{-1}, ν(C=C) 1593-1544 cm^{-1},δ(C-N) 1450 cm^{-1}, ν(C-N) 1355-1300 cm^{-1}, δ(C-H) 889-732 cm^{-1}.

Figure S4–S6 (HQ-S)

Figure S4: ^{1}H NMR spectra of HQ-S (400 MHz, DMSO-d_6)

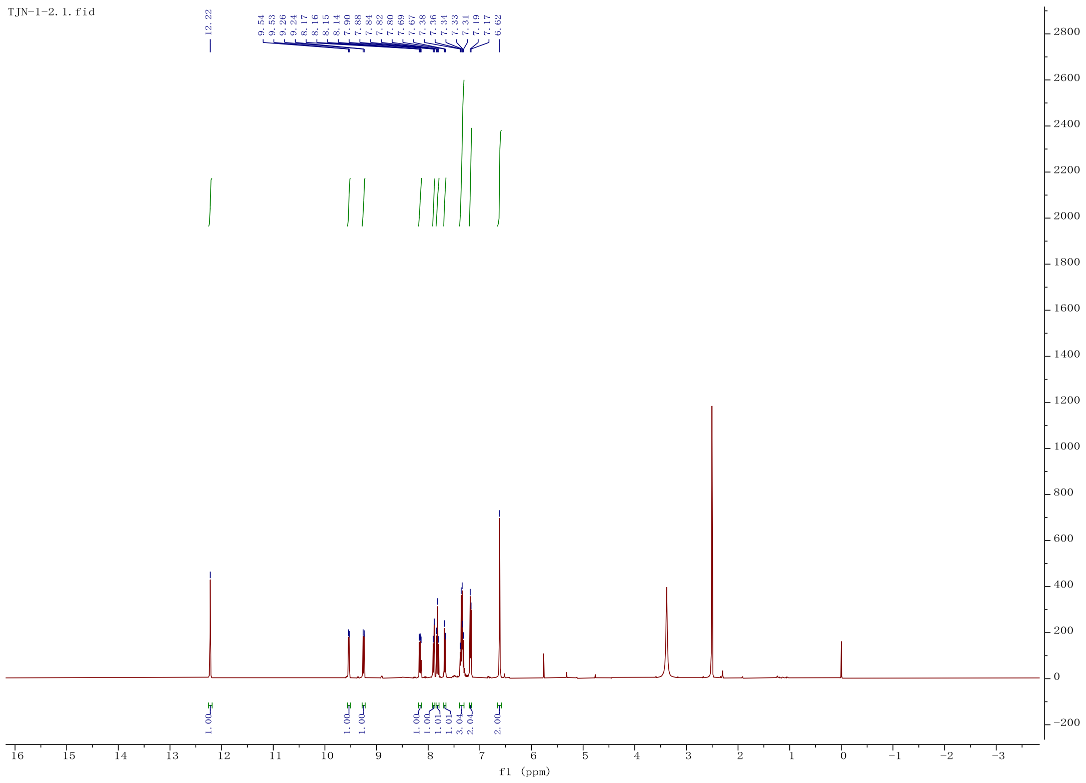

(HQ-S) ^{1}H NMR (400 MHz, DMSO-d6, TMS) δ ppm： 12.22(s, 1H), 9.54 (d, J = 5.9 Hz, 1H), 9.25 (d, J = 8.4 Hz, 1H), 8.15 (dd, J = 8.4, 5.7 Hz, 1H), 7.89 (dd, J = 8.1,1.4 Hz, 1H), 7.82 (t, J = 7.9 Hz,1H), 7.68 (dd, J = 7.8, 1.4 Hz, 1H), 7.34 (dd, J = 11.7, 7.2 Hz, 3H), 7.18 (d, J = 6.8 Hz, 2H), 6.62 (s, 2H).

Figure S5: ^{13}C NMR spectra of HQ-S (100 MHz, DMSO-d_6)

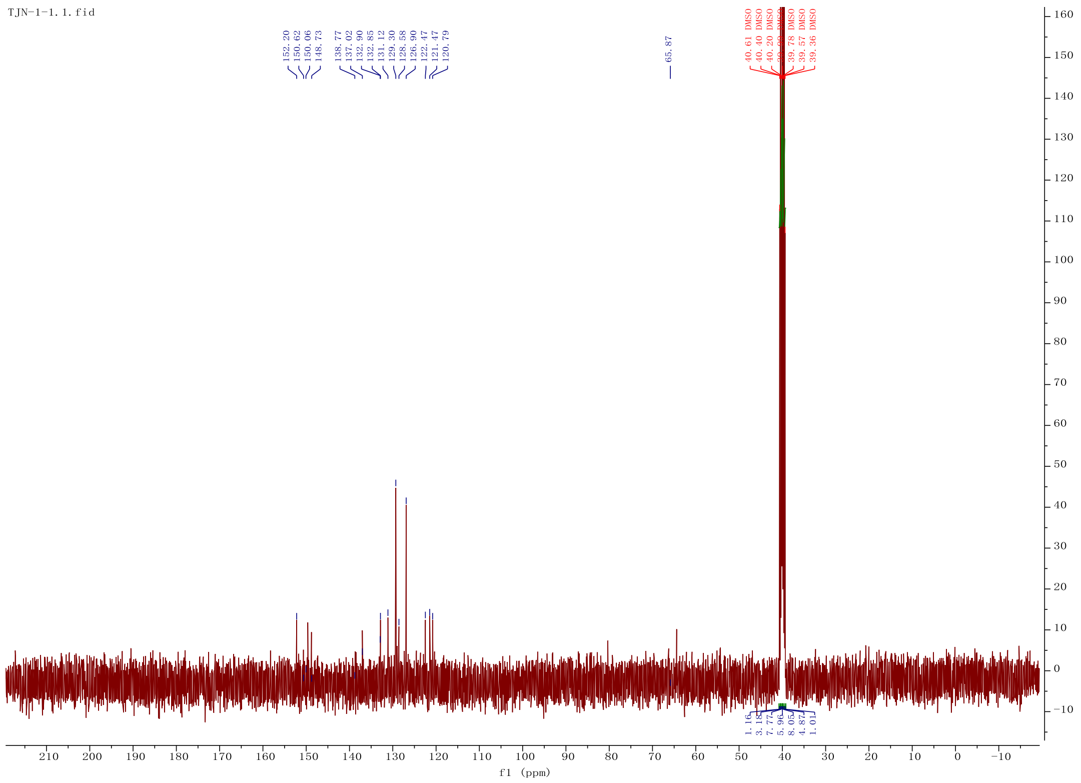

(HQ-S)^{13}C NMR (100 MHz, DMSO-d6) δ ppm 152.2, 150.6, 150.0, 148.7, 137.0, 132.9, 132.8, 131.1, 129.3, 128.6, 126.9, 122.5, 121.5, 120.8, 65.87.

Figure S6: FT-IR spectra of HQ-S

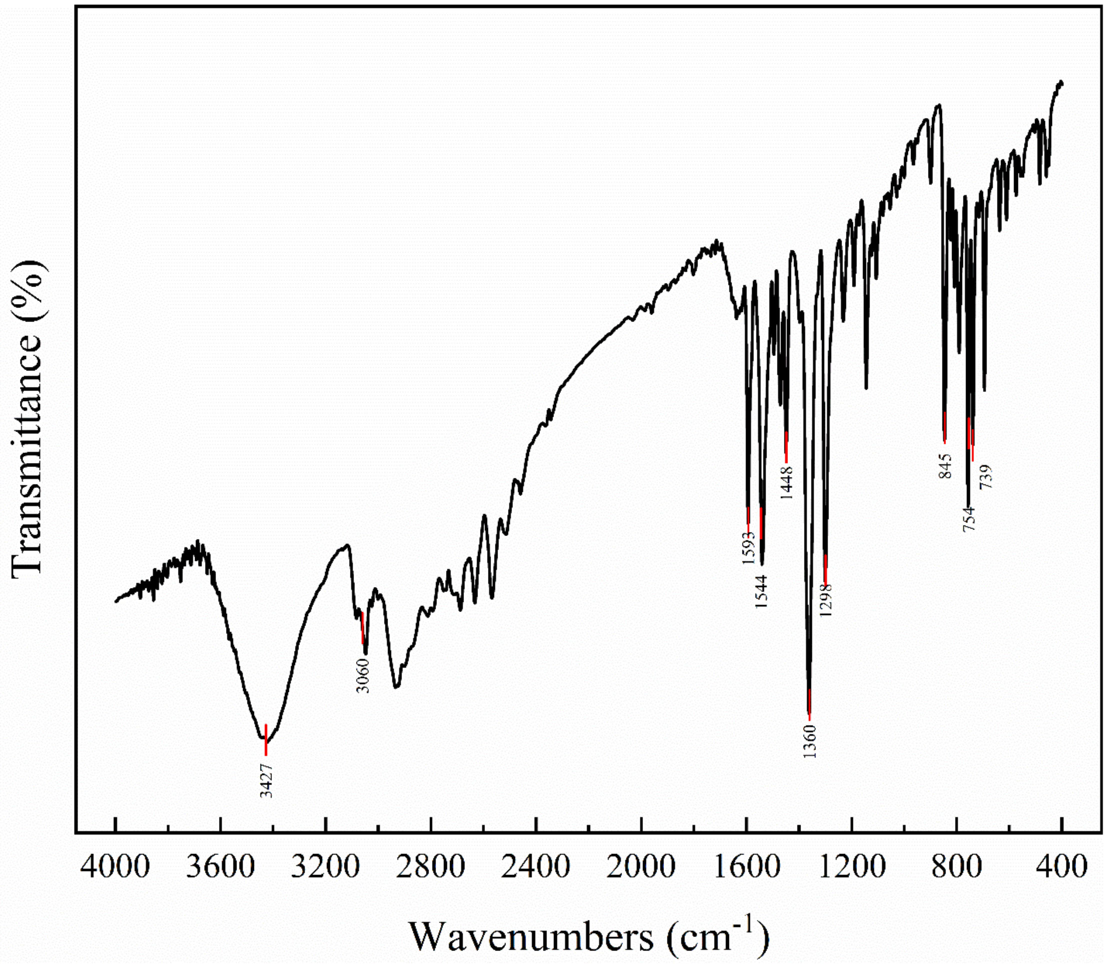

(HQ-S) FTIR ν(-OH), 3427 cm^{-1}, ν(C-H) 3060 cm^{-1}, ν(C=C) 1593-1544 cm^{-1},δ(C-N) 1360-1298 cm^{-1}, ν(C-N) 1448cm^{-1}, δ(C-H) 845-739 cm^{-1}.

Figure S7-S9 (OCH_3-S)

Figure S7: ^{1}H NMR spectra of OCH_3-S (400 MHz, DMSO-d_6)

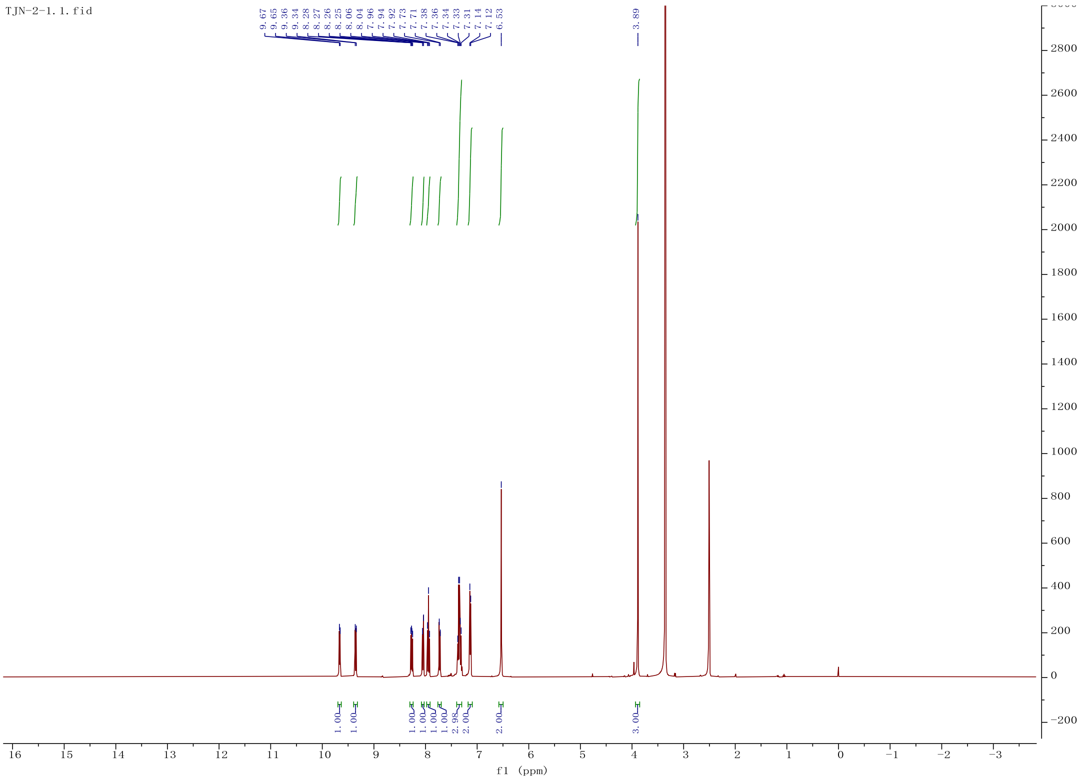

(OCH_3-S) ^{1}H NMR (400 MHz, DMSO-d6, TMS) δ ppm： 9.66 (dd, J = 5.9, 1.5 Hz, 1H), 9.35 (dd J= 8.4, 1.4 Hz, 1H), 8.27 (dd, J =8.4, 5.8 Hz, 1H), 8.05 (d J =8.1 Hz, 1H), 7.94 (t, J = 8.0 Hz, 1H), 7.72 (d, J=8.0 Hz, 1H), 7.40-7.28 (m, 3H), 7.13 (d, J = 6.7 Hz, 2H), 6.53 (s, 2H), 3.89 (s, 3H).

Figure S8: ^{13}C NMR spectra of OCH_3-S (100 MHz, DMSO-d_6)

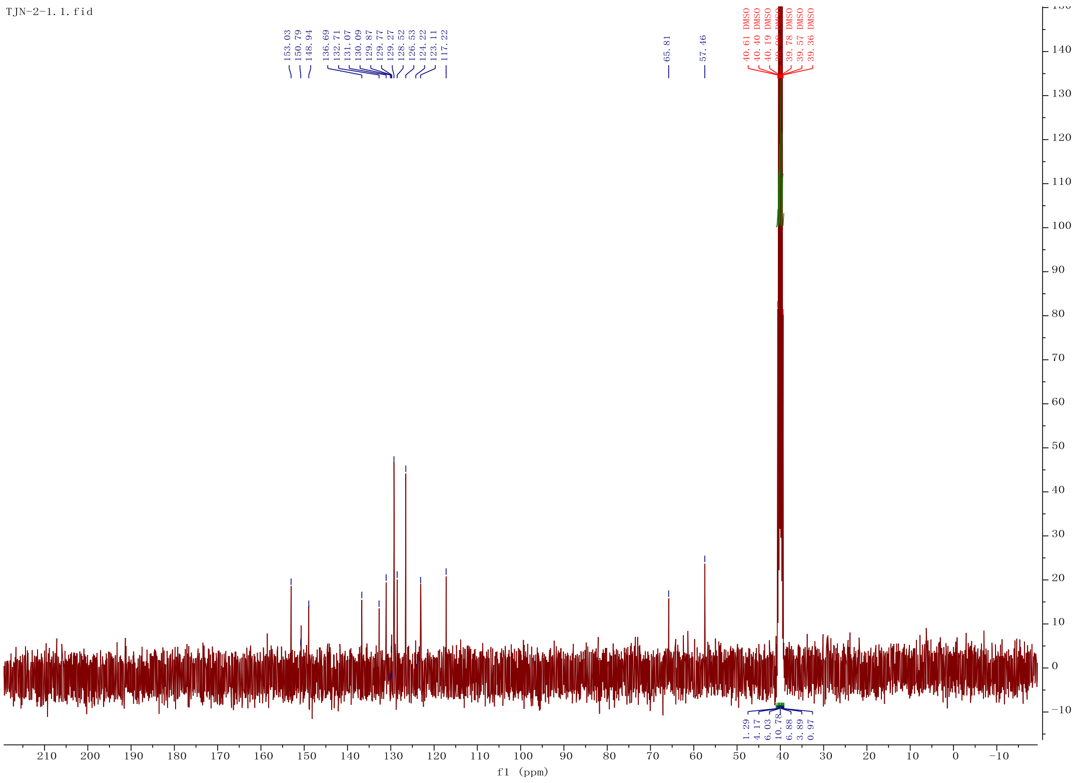

(OCH3-S) 13C NMR (100 MHz, DMSO-d6) δ ppm 153.0, 150.8, 148.9, 136.7, 132.7, 131.0, 130.1, 129.9, 129.8, 129.3, 128.5, 126.5, 124.2, 123.1, 117.2, 65.8, 57.5.

Figure S9: FT-IR spectra of OCH_3-S

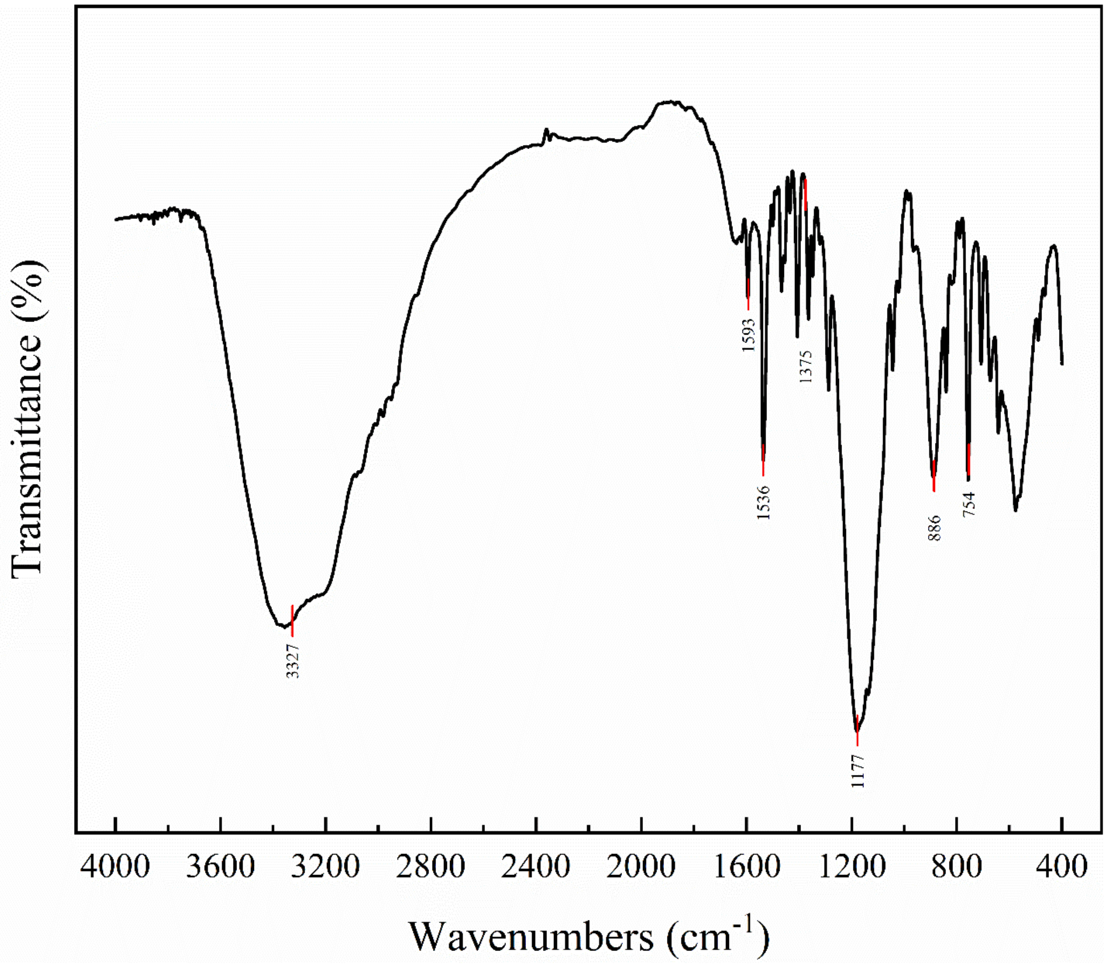

(OCH_3-S) FTIR: ν(-OH), 3327 cm^{-1}, ν(C=C) 1593-1536 cm^{-1}, δ(C-N) 1375 cm^{-1}, ν(C-N) 1177 cm^{-1}, δ(C-H) 886-754 cm^{-1}.
